# Nonlinear association between atherogenic index of plasma and type 2 diabetes mellitus in overweight and obesity patients: evidence from Chinese medical examination data

**DOI:** 10.1186/s12933-024-02330-y

**Published:** 2024-06-29

**Authors:** Yongbing Sun, Fengli Li, Yang Zhou, Ao Liu, Xinbei Lin, Zhi Zou, Xue Lv, Jing Zhou, Zhonglin Li, Xiaoling Wu, Shewei Dou, Michael Zhang, Jiadong Zhu, Yalong Chen, Xinguang Xiao, Yangxi Hu, Hao Li, Yongli Li

**Affiliations:** 1 Department of Medical Imaging, People’s Hospital of Zhengzhou University, #7 Wei Wu Road, Zhengzhou, 450003 Henan China; 2 Department of Bariatric Metabolic Surgery, Central Hospital of Zhengzhou University, #195 Tongbai Road, Zhengzhou, 450003 Henan China; 3 Henan Provincial People’s Hospital, #7 Wei Wu Road, Zhengzhou, 450003 Henan China; 4 Henan Provincial Research Center of Clinical Medicine of Nephropathy, Henan Provincial People’s Hospital, Zhengzhou University People’s Hospital, Henan University People’s Hospital, #7 Wei Wu Road, Zhengzhou, 450003 China; 5 Department of Nuclear Medicine, Henan Provincial People’s Hospital, Zhengzhou, 450003 Henan China; 6 Sevenoaks Health Management Center, Canada-Canada Institute of Health Engineering, University of Manitoba, Winnipeg, Canada; 7 Chronic Health Management Laboratory, Department of Health Management, Henan Provincial People’s Hospital, Zhengzhou, 450003 Henan China; 8 Department of Medical Imaging, Central Hospital of Zhengzhou University, #195 Tongbai Road, Zhengzhou, 450003 Henan China; 9 Fuwaihua Central Vascular Disease Hospital, #1 Fuwai Avenue, Zhengzhou, 451464 Henan China

**Keywords:** Atherogenic index of plasma, Obesity, Type 2 diabetes mellitus, Chinese

## Abstract

**Background:**

The atherogenic index of plasma (AIP) is closely associated with the onset of diabetes, with obesity being a significant risk factor for type 2 diabetes mellitus (T2DM). However, the association between the AIP and T2DM in overweight and obese populations has been infrequently studied. Therefore, this study aimed to explore this association in overweight and obese individuals with T2DM.

**Methods:**

This cross-sectional analysis utilized data from 40,633 participants with a body mass index (BMI) ≥ 24 kg/m^2^ who were screened from January 2018 to December 2023 at Henan Provincial People’s Hospital. Participants were categorized into groups of overweight and obese individuals with and without diabetes according to the T2DM criteria. The AIP, our dependent variable, was calculated using the formula log10 [(TG mol/L)/HDL-C (mol/L)]. We investigated the association between the AIP and T2DM in overweight and obese individuals using multivariate logistic regression, subgroup analysis, generalized additive models, smoothed curve fitting, and threshold effect analysis. Additionally, mediation analysis evaluated the role of inflammatory cells in AIP-related T2DM.

**Results:**

Overweight and obese patients with T2DM exhibited higher AIP levels than those without diabetes. After adjusting for confounders, our results indicated a significant association between the AIP and the risk of T2DM in overweight and obese individuals (odds ratio (OR) = 5.17, 95% confidence interval (CI) 4.69–5.69). Notably, participants with a high baseline AIP (Q4 group) had a significantly greater risk of T2DM than those in the Q1 group, with an OR of 3.18 (95% CI 2.94–3.45). Subgroup analysis revealed that the association between the AIP and T2DM decreased with increasing age (interaction *P* < 0.001). In overweight and obese populations, the association between AIP and T2DM risk displayed a J-shaped nonlinear pattern, with AIP > – 0.07 indicating a significant increase in T2DM risk. Various inflammatory cells, including neutrophils, leukocytes, and monocytes, mediated 4.66%, 4.16%, and 1.93% of the associations, respectively.

**Conclusion:**

In overweight and obese individuals, the AIP was independently associated with T2DM, exhibiting a nonlinear association. Additionally, the association between the AIP and T2DM decreased with advancing age. Multiple types of inflammatory cells mediate this association.

**Supplementary Information:**

The online version contains supplementary material available at 10.1186/s12933-024-02330-y.

## Introduction

Type 2 diabetes mellitus (T2DM) poses a significant global public health challenge and is characterized by a complex interaction of endocrine-metabolic factors driven by chronic excessive energy intake [1]. Recent statistics indicate that a substantial number of 529 million people worldwide were affected by T2DM in 2021, and this number is projected to rise to approximately 1.31 billion by 2050 [2]. Notably, China has the largest population of individuals with diabetes, with 140 million affected individuals in 2021 [3]. Over the past two decades, the global prevalence of T2DM has doubled, mainly due to the obesity epidemic [4]. The effect of a high body mass index (BMI) on disability or mortality among individuals with T2DM increased by 24.3% worldwide between 1990 and 2021 [2]. However, diabetes is largely preventable, and in some cases, it can be reversed if it is detected and managed early in the disease course. Therefore, identifying an easily accessible biomarker in clinical practice may contribute to the primary prevention and early identification of at-risk populations, as well as facilitate the development of effective primary prevention strategies. This is crucial in addressing the increasing prevalence of diabetes in overweight and obese populations.

The atherogenic index of plasma (AIP) is a robust marker for evaluating atherosclerosis and severe cardiovascular events. It is calculated as the logarithm of the ratio of triglycerides (TG) to high-density lipoprotein cholesterol (HDL-C) [5, 6]. In 2001, Dobiásová and Frohlich proposed the AIP as an improved indicator of blood lipids [7]. Due to its affordability and accessibility, the AIP has been utilized in the treatment of various conditions, including hypertension [8], obesity [9], and metabolic syndrome [10]. Studies have also discovered a strong association between AIP and T2DM [11, 12], with higher AIP levels indicating an increased risk of developing T2DM. Research on the correlation between AIP and T2DM has extensively examined individuals with different glycemic statuses, such as those with coronary heart disease [13], nonalcoholic fatty liver disease [14], prediabetes [5], and American adults diagnosed with T2DM in the National Health and Nutrition Examination Survey (NHANES) database [11]. Although obesity is a principal cause of insulin resistance among diabetic patients and an independent risk factor for T2DM and its complications, as well as all-cause mortality [15, 16], the association between the AIP and T2DM, particularly in individuals who are overweight and obese, has not been sufficiently studied. Additionally, there is a lack of large-scale demographic evidence from China.

Inflammation plays a crucial role in the pathogenesis of T2DM, causing inflammation in pancreatic β-cells and exacerbating insulin resistance [17–19]. Atherogenic lipids associated with obesity further contribute to a low-grade inflammatory state through lipotoxic effects and the release of cytokines [20]. Considering the significant role of inflammation in the development of T2DM, this study aims to investigate the association between AIP and T2DM in overweight and obese individuals, as well as evaluate its predictive ability for T2DM risk. Additionally, the study seeks to explore the involvement of inflammatory cells in mediating the association between AIP and T2DM.

## Materials and methods

### Individuals and the criteria for inclusion

The research data were collected from the medical records of individuals who underwent health screenings at the Health Management Center of Henan Provincial People’s Hospital from January 2018 to December 2023. The study received ethical approval from the Ethics Committee of Henan Provincial People’s Hospital (Approval Code: 2021 Lunar Review No. 68), and informed consent was waived for all participants. The dataset was registered on clinicaltrials.gov (Registration Code: NCT03699228) and is associated with the China Health Quantitative CT Big Data Research Project Group.

Participants were selected based on specific criteria. These criteria included: (1) age between 20 and 80 years, (2) BMI of ≥ 24 kg/m^2^, (3) completed general and questionnaire information, and (4) completed fasting plasma glucose (FPG) and glycosylated hemoglobin (HbA1c) levels. The exclusion criteria were as follows: (1) a history of cancer, psychiatric or cognitive disorders in women, mobility impairments, pregnancy, or breastfeeding, (2) previous or current pancreas-related disorders, (3) extreme values in test results, (4) previous or current other metabolic disorders such as primary aldosteronism, pheochromocytoma, liver injury, or abnormal thyroid function, (5) unknown diabetic status, and (6) history of antibiotic use in the last two weeks.

**Fig. 1:**
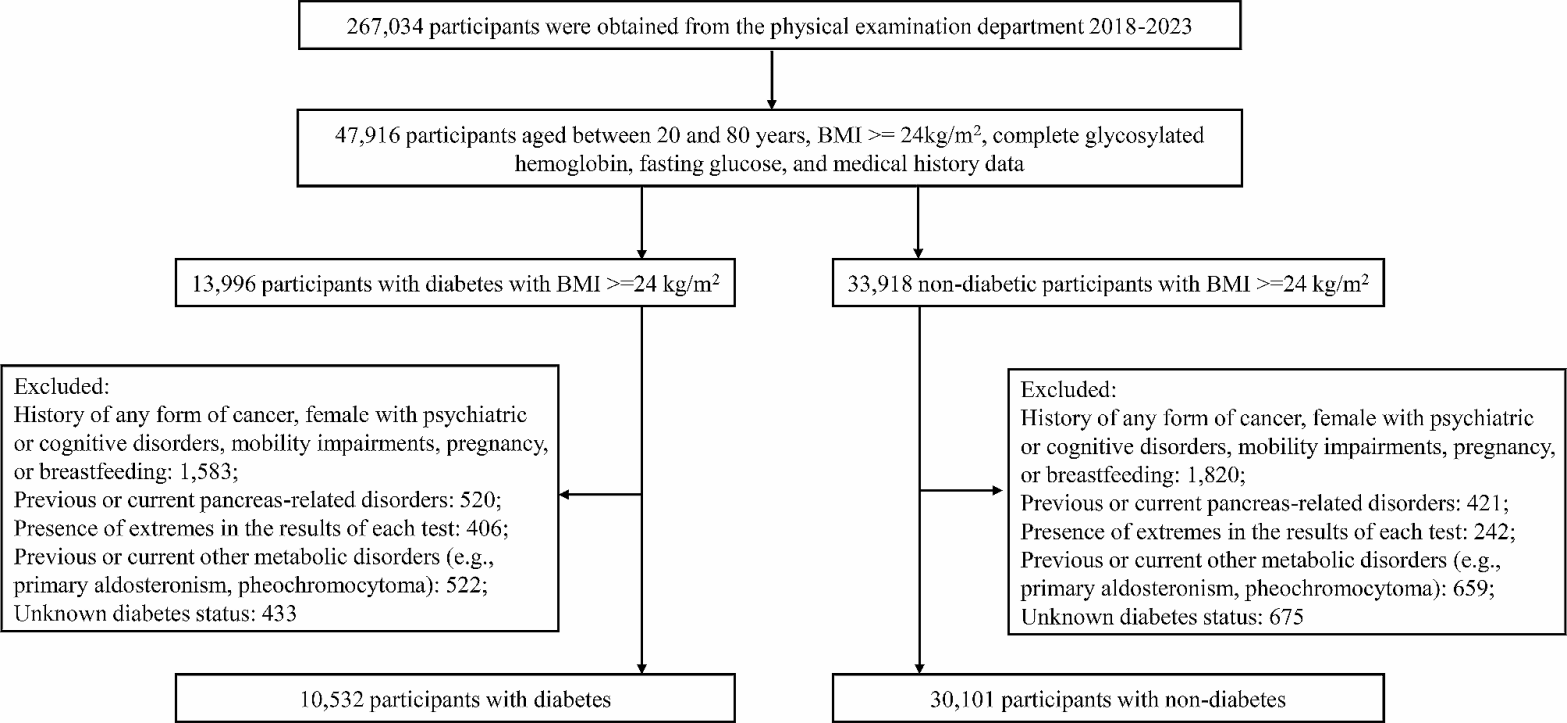
Flowchart of participants selection

Initially, a total of 267,034 participants were included. However, only 47,916 participants met the criteria for further analysis, which included a BMI of ≥ 24 kg/m^2^ and complete glycated hemoglobin, fasting glucose, and medical history data. These participants were then divided into diabetic (13,996 patients) and nondiabetic (33,918 patients) groups based on T2DM diagnostic criteria and screened for clinical data completeness. Ultimately, 40,633 participants (10,532 in the diabetic group and 30,101 in the nondiabetic group) were included in the final analysis. The comprehensive data collected by a professional researcher included age, sex, ethnicity, medical history, and medication history. The participant screening process is illustrated in Fig. 1.

### Definitions of the exposure and outcome variables

The exposure variable, AIP, was calculated as log10[(TG mol/L)/HDL-C (mol/L)] [5]. The outcome variable was overweight and obese T2DM, which was defined according to the American Diabetes Association’s diagnostic criteria: self-reported diagnosis, use of insulin or oral hypoglycemic medication, HbA1c ≥ 6.5%, or FPG ≥ 7 mmol/L [21].

BMI was calculated as weight divided by height squared (kg/m^2^) and categorized according to the Chinese national standard into thresholds for overweight (24 ≤ BMI < 27.9 kg/m^2^) and obesity (BMI ≥ 28 kg/m^2^) [22].

Hypertension was defined as systolic blood pressure ≥ 140 mmHg or diastolic blood pressure ≥ 90 mmHg, self-reported hypertension, antihypertensive medication use, or antihypertensive therapy [23]. Previous studies have used cutoff points of 60 and 80 to divide total serum protein into three groups. Elevated levels of alanine aminotransferase (ALT), aspartate transaminase (AST), and glutamyl transpeptidase (GGT) were defined as values exceeding 40 U/L, and these cutoff points were used to categorize ALT, AST, and GGT into two groups. Alkaline phosphatase (ALP) was divided into three groups using cutoff points of 40 and 150 [24]. Total bilirubin, creatinine (Cre), and uric acid (UA) were then divided into three groups based on tertiles.

### Laboratory measurements

Prior to conducting the surveys, all researchers underwent standardized training to ensure the impartiality and accuracy of the data. A standardized questionnaire was utilized to collect the relevant data from participants, including their medical history, such as previous or current diabetes, various cancers, endocrine disorders, and recent medication use within the past two weeks. Once the questionnaire was completed, the researchers organized, summarized, and verified the data.

At 8 a.m., following an overnight fast, venous blood samples were obtained from the participants to measure various biochemical markers, including total protein, total bilirubin, ALT, AST, GGT, ALP, Cre, UA, total cholesterol (TC), low-density lipoprotein cholesterol (LDL-C), TG, HDL-C, FPG, and HbA1c. Blood glucose levels were measured using an Olympus^®^ AU 5400 automated biochemistry analyzer (Olympus Corporation, Japan, Shizuoka Prefecture). Standard laboratory procedures were followed for evaluating the remaining indicators. To measure diastolic and systolic blood pressure (SBP and DBP), the research staff employed an electronic sphygmomanometer (Omron Company, OMRON U30, Kyoto, Japan). The right arm was positioned in a semiflexed posture at heart level during the measurement.

### Potential covariates

Covariate data were collected as follows: (1) demographic data, including sex, ethnicity, occupation, and age; (2) physical examination parameters, including BMI, SBP, and DBP; (3) medical conditions and hypertension; and (4) laboratory indicators, including total protein, total bilirubin, ALT, AST, GGT, ALP, Cre, and UA.

### Statistical analysis

The statistical analysis was conducted using R, version 4.2.0 (R Foundation), in conjunction with EmpowerStats (http://www.empowerstats.com, X&Y Solutions, Inc., Boston, MA). A significance level of *P* < 0.05 was employed for all statistical tests.

For each dataset, a normality test was performed to identify continuous variables. Normally distributed continuous variables are presented as the mean ± standard deviation, whereas skewed continuous variables are described as the median (upper and lower interquartile range). Between-group differences were assessed using t tests or rank sum tests. Categorical variables are presented as frequencies with accompanying percentages, and comparisons were conducted using the chi-square test or Fisher’s exact test.

Initially, the effect of each variable on the risk of T2DM was assessed using univariate logistic regression models. ORs and corresponding 95% confidence intervals (CIs) were computed. To account for potential confounding variables, a multivariate logistic regression analysis with stepwise regression was performed. Variables with a variance inflation factor (VIF) > 10 were excluded from the analysis to investigate the association between the AIP and the risk of T2DM in overweight and obese individuals. The crude model did not include any adjustments for covariates. Model I was adjusted for age, sex, and ethnicity, whereas Model II included adjustments for all covariates, including age, sex, occupation, BMI, hypertension, total protein, total bilirubin, ALT, AST, GGT, ALP, Cre, and UA individually. Moreover, the AIP was categorized into quartiles to form the basis of the final model, ensuring the reliability of the results. The model assessed the association between quartiles and the risk of diabetes, using the lowest quartile as the reference category. Subsequently, a generalized additive model based on smoothed curve fitting was used to visualize the dose-response association between the AIP and the risk of T2DM in overweight and obese patients. A two-stage logistic regression model was constructed by analyzing data on both sides of the inflection point to explore potential nonlinear associations. The log-likelihood ratio was employed to select the most appropriate model for describing the association between the AIP and the risk of T2DM in overweight and obese patients. Stratified analyses and interaction tests were also performed based on Model II to investigate other factors influencing the association between the AIP and the development of T2DM. Additionally, causal mediation analysis was conducted to examine whether the association between ALP and the risk of T2DM in overweight and obese individuals is mediated by inflammatory cells.

## Results

### Baseline details about the participants

**Fig. 2:**
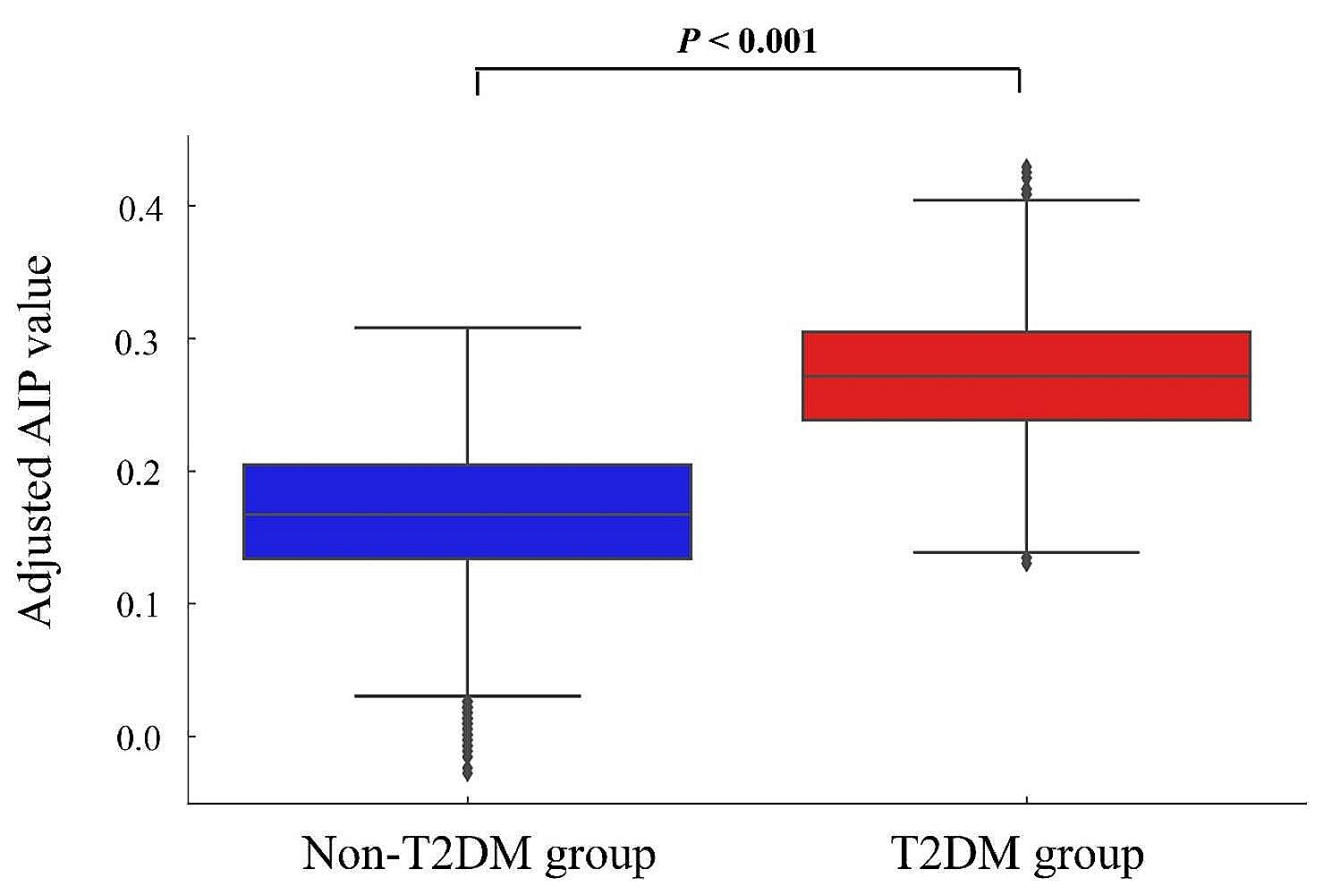
Age-adjusted AIP box plots for overweight and obese non-T2DM and T2DM groups. The box indicates the interquartile range (IQR) and the center line indicates the median. Whiskers above and below the box indicate the data range and dots indicate outliers. *T2DM* type 2 diabetes mellitus, *AIP* atherogenic index of plasma. *P* < 0.001, as compared with non-T2DM group

There was a total of 40,633 participants in this study who were either overweight or obese. Among them, 10,532 had diabetes, and 30,101 did not have diabetes. The prevalence of diabetes in the study population was determined to be 25.92%. After adjusting for the age variable, AIP values were significantly higher in overweight and obese individuals with T2DM compared to those without T2DM (Fig. 2). Table 1 provides an overview of the baseline characteristics of the study population, categorized by diabetes diagnosis. The overweight and obese group with T2DM consisted primarily of males, staff members, and individuals with hypertension. Additionally, participants with T2DM had higher values of various indicators, including age, BMI, total protein, ALT, AST, GGT, ALP, TC, TG, FPG, HbA1c, AIP, lymphocytes, monocytes, eosinophils, basophils, neutrophils, and white blood cells (WBC). Conversely, T2DM participants exhibited lower levels of total bilirubin, Cre, UA, LDL-C, and HDL-C compared to nondiabetic participants. These differences were all statistically significant (*P* < 0.05).

**Table 1:** Baseline characteristics of overweight and obese participants by T2DM status

Variables	Overall	Non-T2DM	T2DM	*P* value
N	40,633	30,101	10,532	
Sex, n (%)				< 0.001
Female	11,050 (27.19)	7914 (26.29)	3136 (29.78)	
Male	29,583 (72.81)	22,187 (73.71)	7396 (70.22)	
Ethnic group, n (%)				0.935
Non-han	637 (1.57)	471 (1.56)	166 (1.58)	
Han	39,996 (98.43)	29,630 (98.44)	10,366 (98.42)	
Occupation, n (%)				< 0.001
Unemployment	13,745 (33.83)	11,193 (37.18)	2552 (24.23)	
Doctors	817 (2.01)	290 (0.96)	527 (5.00)	
Staff members	26,071 (64.16)	18,618 (61.85)	7453 (70.77)	
Age, years	49.61 ± 12.54	47.17 ± 11.90	56.60 ± 11.64	< 0.001
BMI, kg/m^2^	27.15 ± 2.58	27.00 ± 2.46	27.60 ± 2.86	< 0.001
Hypertension, n (%)				< 0.001
No	26,608 (65.48)	21,503 (71.44)	5105 (48.47)	
Yes	14,025 (34.52)	8598 (28.56)	5427 (51.53)	
Total protein, g/L	71.97 ± 4.25	71.90 ± 4.06	72.17 ± 4.75	< 0.001
Total bilirubin, µmol/L	12.25 ± 5.59	12.34 ± 5.52	11.99 ± 5.79	< 0.001
ALT, U/L	23.40 (16.90, 34.50)	22.76 (16.80, 34.40)	23.40 (17.00, 34.70)	< 0.001
AST, U/L	21.00 (17.50, 26.10)	20.70 (17.70, 25.90)	21.02 (16.90, 26.70)	< 0.001
GGT, U/L	29.20 (20.30, 46.10)	28.50 (19.70, 44.80)	31.30 (22.00, 50.30)	< 0.001
ALP, U/L	72.38 ± 20.84	70.46 ± 19.12	77.87 ± 24.31	< 0.001
Cre, µmol/L	69.52 ± 20.12	70.30 ± 17.85	67.27 ± 25.36	< 0.001
UA, µmol/L	361.14 ± 92.06	369.48 ± 91.28	337.32 ± 90.10	< 0.001
TC, mmol/L	4.95 ± 1.00	4.85 ± 0.93	4.96 ± 1.18	0.007
LDL-C, mmol/L	2.91 ± 0.77	2.93 ± 0.74	2.86 ± 0.86	< 0.001
TG, mmol/L	1.76 (1.26, 2.55)	1.69 (1.22, 2.41)	2.00 (1.40, 2.95)	< 0.001
HDL-C, mmol/L	1.21 ± 0.26	1.23 ± 0.26	1.16 ± 0.25	< 0.001
FPG, mmol/L	5.91 ± 1.93	5.09 ± 0.55	8.24 ± 2.48	< 0.001
HbA1c, %	6.07 ± 1.05	5.58 ± 0.40	7.49 ± 1.04	< 0.001
AIP	0.17 (–0.01 to 0.36)	0.15 (– 0.03, 0.34)	0.24 (0.06, 0.44)	< 0.001
Lymphocytes (1000 cells/µL)	2.05 ± 0.68	2.03 ± 0.69	2.11 ± 0.65	< 0.001
Monocytes (1000 cells/µL)	0.42 ± 0.14	0.41 ± 0.14	0.44 ± 0.15	< 0.001
Eosinophils (1000 cells/µL)	0.12 (0.08, 0.20)	0.12 (0.08, 0.19)	0.13 (0.08, 0.20)	< 0.001
Basophils (1000 cells/µL)	0.40 (0.30, 0.60)	0.39 (0.30, 0.60)	0.40 (0.20, 0.60)	< 0.001
Neutrophils (1000 cells/µL)	3.61 ± 1.16	3.52 ± 1.11	3.85 ± 1.25	< 0.001
WBC (1000 cells/µL)	6.26 ± 1.60	6.15 ± 1.55	6.59 ± 1.67	< 0.001

Normally distributed continuous variables are presented as the mean ± standard deviation, whereas skewed continuous variables are described as the median (upper and lower interquartile range), categorical variables are presented as frequencies with accompanying percentages

*BMI* body mass index, *ALT* alanine aminotransferase, *AST* aspartate transaminase, *GGT* glutamyl transpeptidase, *ALP* Alkaline phosphatase, *Cre* Creatinine, *UA* Uric acid, *TC* total cholesterol, *LDL-C* low-density lipoprotein cholesterol, *TG* triglycerides, *HDL-C* high-density lipoprotein cholesterol, *FPG* fasting plasma glucose, *HbA1c* Glycosylated haemoglobin, *AIP* atherogenic index of plasma, *WBC* White blood cell

### Associations between the AIP and overweight and obese T2DM individuals according to the different models

Table 2 presents the results of the univariate logistic regressions, which were employed to assess the significance of the traditional risk factors and select covariates for the subsequent multivariate regression analyses. Among these factors, doctors (with unemployment as the reference group), Hypertension (with non-hypertension as the reference group), and AIP were found to be the most significant variables for increased risk of T2DM. Ethnicity was excluded as a covariate in the multivariate logistic regression analysis. This analysis further confirmed the independent association between the AIP score and T2DM status. As depicted in Table 3, both Model I (odds ratio (OR) = 5.85, 95% CI 5.37–6.37, *P* < 0.001) and Model II (OR = 5.17, 95% CI 4.69–5.69, *P* < 0.001) demonstrated a significant association between continuous AIP and T2DM, even after adjusting for confounding factors. Specifically, for every 1-unit increase in the AIP, the risk of T2DM increased by 5.17 (*P* < 0.001). To facilitate the analysis, the continuous variables were categorized using quartiles of the AIP. After adjusting for multiple confounders, it was found that the risk of T2DM was 3.18 times higher in the group with the highest AIP compared to the group with the lowest AIP (*P* < 0.001).

**Table 2:** Univariate logistic analysis for predicting overweight and obese T2DM

Variables	Statistics	OR (95% CI)	*P* value
Sex, *n* (%)			
Female	11,050 (27.19)	1.0	
Male	19,583 (72.81)	0.84 (0.80,0.88)	< 0.001
Ethnic group, n (%)			
Non-han	637 (1.57)	1.0	
Han	39,996 (98.43)	0.99 (0.83, 1.19)	0.935
Occupation, n (%)			
Unemployment	13,745 (33.83)	1.0	
Doctors	817 (2.01)	7.97 (6.86, 9.26)	< 0.001
Staff members	26,071 (64.16)	1.76 (1.67, 1.85)	< 0.001
Age, years	49.61 ± 12.54	1.07 (1.07, 1.07)	< 0.001
BMI, kg/m^2^	27.15 ± 2.58	1.09 (1.08, 1.10)	< 0.001
Hypertension, n (%)			
No	26,608 (65.48)	1.0	
Yes	14,025 (34.52)	2.66 (2.54, 2.78)	< 0.001
Total protein, g/L	71.97 ± 4.25	1.01 (1.01, 1.02)	< 0.001
Total bilirubin, µmol/L	12.25 ± 5.59	0.99 (0.98, 0.99)	< 0.001
ALT, U/L	23.40 (16.90, 34.50)	1.00 (1.00, 1.00)	< 0.001
AST, U/L	21.00 (17.50, 26.10)	1.00 (1.00, 1.01)	< 0.001
GGT, U/L	29.20 (20.30, 46.10)	1.00 (1.00, 1.00)	< 0.001
ALP, U/L	72.38 ± 20.84	1.02 (1.02, 1.02)	< 0.001
Cre, µmol/L	69.52 ± 20.12	0.99 (0.98, 0.99)	< 0.001
UA, µmol/L	361.14 ± 92.06	1.00 (1.00, 1.00)	< 0.001
TC, mmol/L	4.95 ± 1.00	1.01 (1.00, 1.04)	< 0.001
LDL-C, mmol/L	2.91 ± 0.77	0.88 (0.85, 0.90)	< 0.001
TG, mmol/L	1.76 (1.26, 2.55)	1.18 (1.16, 1.19)	< 0.001
HDL-C, mmol/L	1.21 ± 0.26	0.37 (0.33, 0.40)	< 0.001
FBG, mmol/L	5.91 ± 1.93	13.15 (12.43, 13.91)	< 0.001
HbA1c, %	6.07 ± 1.05	982.16 (799.58, 1206.44)	< 0.001
AIP	0.17 (-0.01, 0.36)	3.26 (3.02, 3.51)	< 0.001

Normally distributed continuous variables are presented as the mean ± standard deviation, whereas skewed continuous variables are described as the median (upper and lower interquartile range), categorical variables are presented as frequencies with accompanying percentages

*BMI* body mass index, *ALT* alanine aminotransferase, *AST* aspartate transaminase, *GGT* glutamyl transpeptidase, *ALP* Alkaline phosphatase, *Cre* Creatinine, *UA* Uric acid, *TC* total cholesterol, *LDL-C* low-density lipoprotein cholesterol, *TG* triglycerides, *HDL-C* high-density lipoprotein cholesterol, *FBG* fasting blood glucose, *HbA1c* Glycosylated hemoglobin. *AIP* atherogenic index of plasma. *OR* odds ratio, *CI* confidence interval

**Table 3:** Relationship between AIP and overweight and obese T2DM in different models

	Crude model		Model I		Model II	
	OR (95% CI)	*P* value	OR (95% CI)	*P* value	OR (95% CI)	*P* value
AIP index	3.26 (3.02, 3.51)	< 0.001	5.85 (5.37, 6.37)	< 0.001	5.17 (4.69, 5.69)	< 0.001
Q1	Reference		Reference		Reference	
Q2	1.33 (1.25, 1.43)	< 0.001	1.40 (1.30, 1.51)	< 0.001	1.33 (1.23, 1.44)	< 0.001
Q3	1.63 (1.53, 1.74)	< 0.001	1.97 (1.83, 2.12)	< 0.001	1.88 (1.74, 2.03)	< 0.001
Q4	2.34 (2.19, 2.49)	< 0.001	3.53 (3.28, 3.79)	< 0.001	3.18 (2.94, 3.45)	< 0.001
***P*** for trend	1.32 (1.29, 1.35)	< 0.001	1.52 (1.48, 1.55)	< 0.001	1.47 (1.44, 1.51)	< 0.001

Crude model: No adjustment for model variables

Model I was adjusted for age, sex, and occupation

Model II was adjusted for all covariates, including age, sex, occupation, BMI, hypertension, total protein, total bilirubin, ALT, AST, GGT, ALP, Cre, and UA

*AIP* atherogenic index of plasma, *OR* odds ratio, *CI* confidence interval

**Fig. 3:**
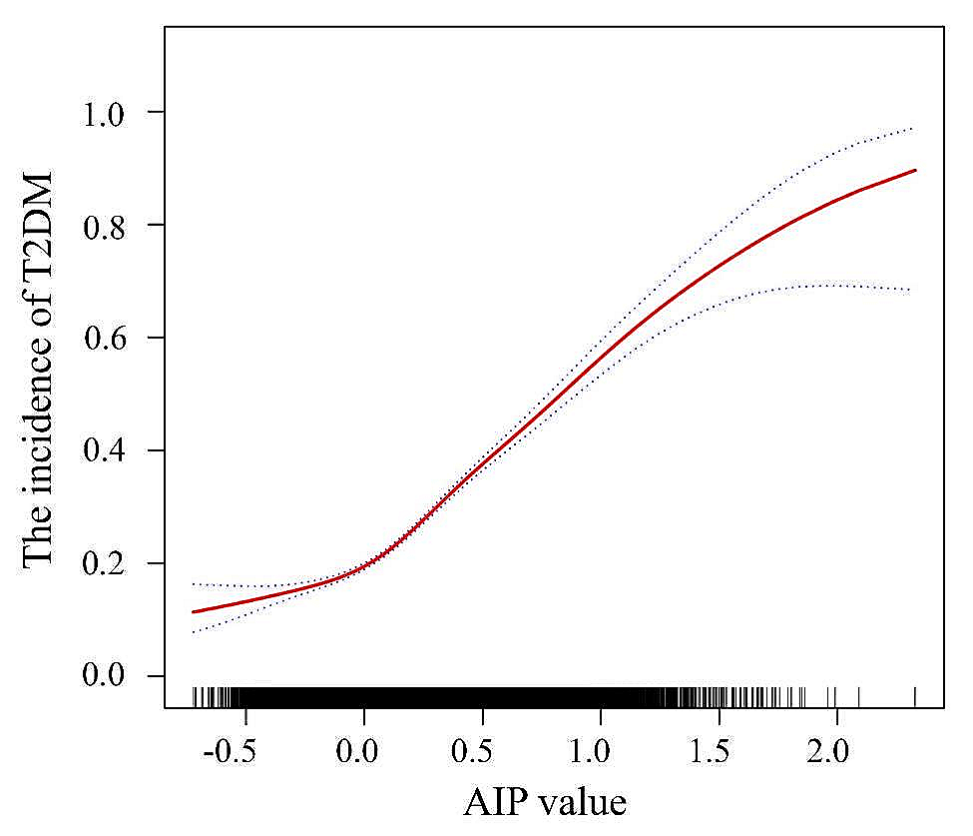
Generalized additive model with fitting smoothness for the dose–response association between AIP and overweight and obese T2DM risk. The red solid line represents the probability of T2DM occurrence, and the blue dotted line represents the 95% CI curve. *T2DM* Type 2 Diabetes Mellitus, *AIP* Atherogenic Index of Plasma. All covariates, including age, sex, occupation, BMI, hypertension, total protein, total bilirubin, ALT, AST, GGT, ALP, Cre, and UA, were adjusted in this model

Moreover, the association between the AIP and T2DM was examined through a smooth curve fitting, revealing a nonlinear association (refer to Fig. 3). The results of the threshold effect analysis can be seen in Table 4. When the AIP was greater than or equal to − 0.07, the risk of T2DM increased by 5.71 times for each one-unit increase in the AIP. Conversely, when the AIP was less than − 0.07, the risk of T2DM increased by 1.93 times for each one-unit increase in the AIP (*P* < 0.05).

**Table 4:** The result of the two-piecewise logistic regression model

	Linear regression		Break point	< K		> K		LLR test
	OR (95% CI)	P value	(K)	OR (95% CI)	P value	OR (95% CI)	P value	P
AIP	5.17 (4.69, 5.69)	< 0.001	1.93 (1.17, 3.18) 0.010	1.93 (1.17, 3.18)	0.010	5.71 (5.12, 6.37)	< 0.001	< 0.001

AIP, atherogenic index of plasma. All covariates including age, sex, occupation, BMI, hypertension, total protein, total bilirubin, ALT, AST, GGT, ALP, Cre, and UA were adjusted in this model. OR, odds ratio; CI, confidence interval.

### Subgroup analysis

**Fig. 4:**
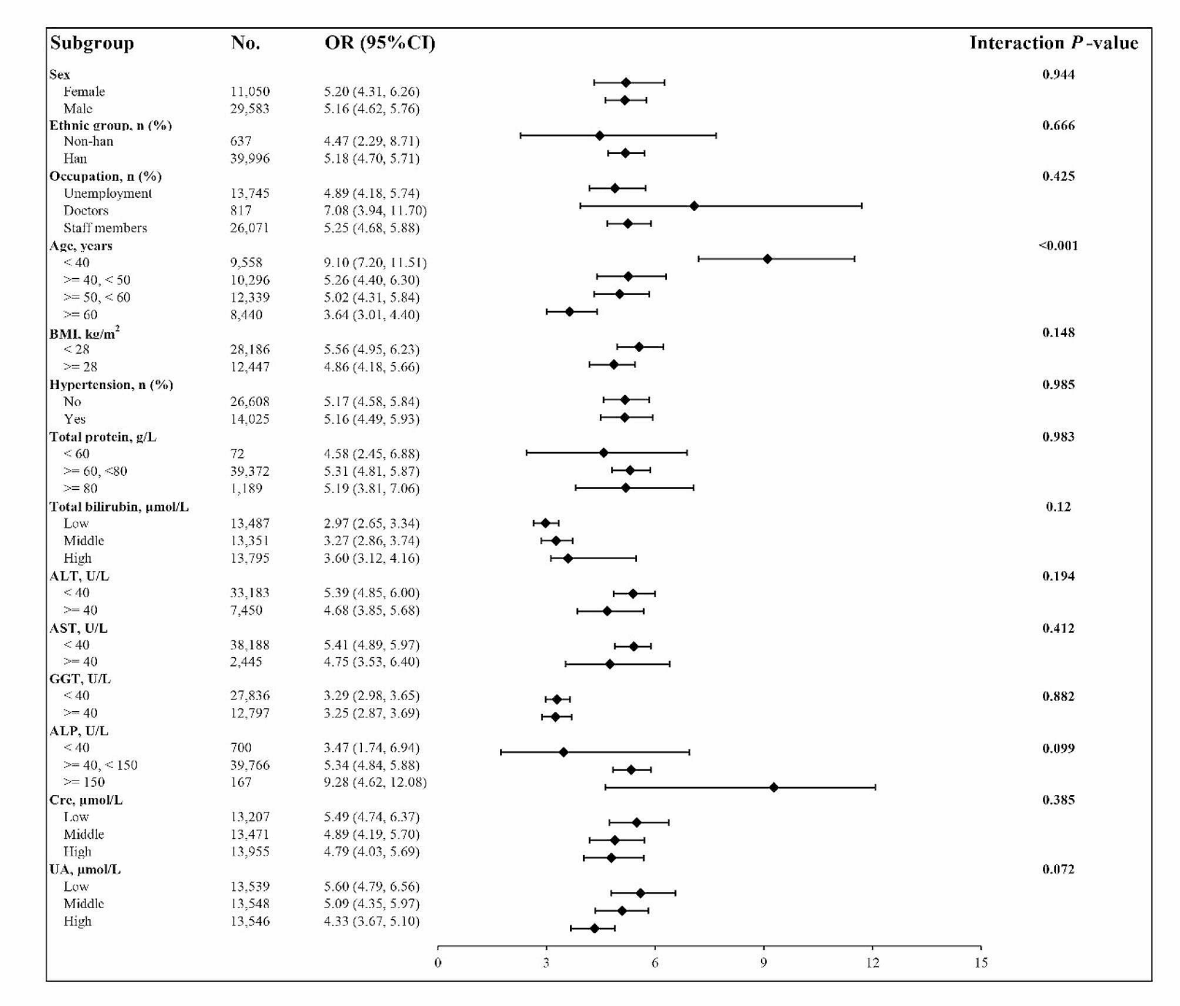
The association between AIP of overweight and obese T2DM according to different subgroups. Adjusted for all covariates except for this subgroup of variables. *BMI* body mass index, *ALT* alanine aminotransferase, *AST* aspartate transaminase, *GGT* glutamyl transpeptidase, *ALP* Alkaline phosphatase, *Cre* Creatinine, *UA* Uric acid, *OR* odds ratio, *CI* confidence interval

**Fig. 5:**
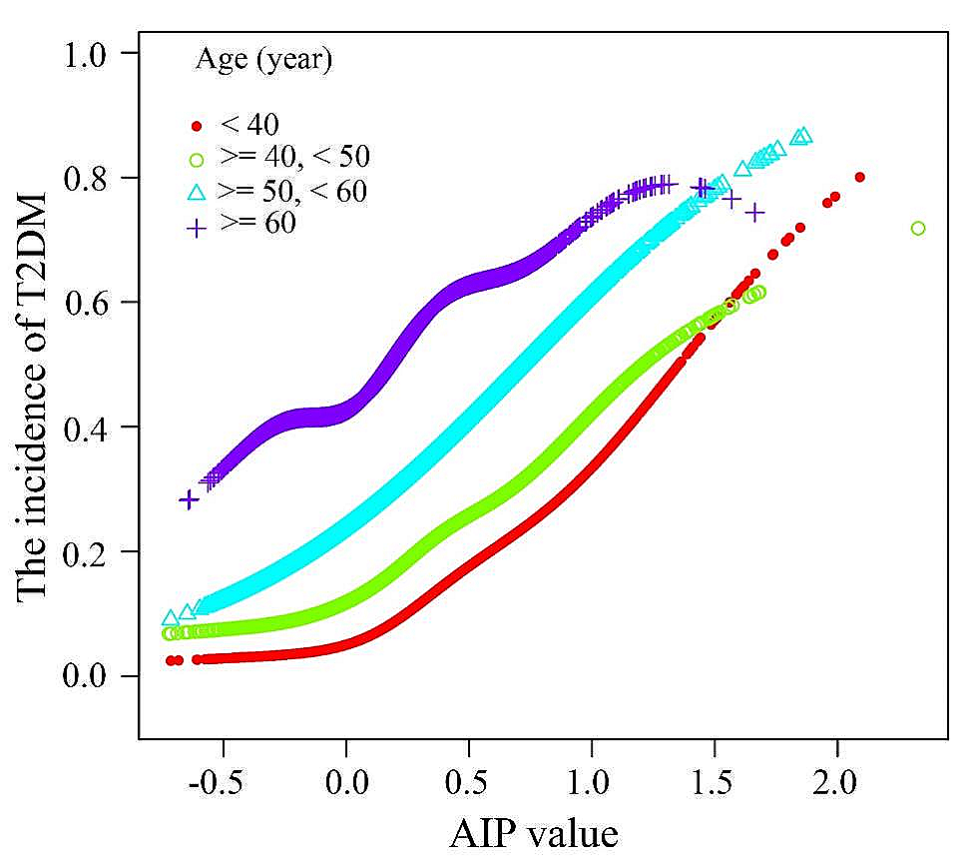
The association between AIP of overweight and obese T2DM according to different age groups. *T2DM* type 2 diabetes mellitus. AIP, atherogenic index of plasma. All covariates including sex, occupation, BMI, hypertension, total protein, total bilirubin, ALT, AST, GGT, ALP, Cre, and UA were adjusted in this model

In addition to age, consistent findings were observed in the subgroup analyses, as shown in Fig. 4. No significant interactions were found when stratified by sex, ethnic group, occupation, BMI, hypertension, total protein, total bilirubin, ALT, AST, GGT, ALP, Cre, or UA (*P* for interaction > 0.05). However, a significant interaction was noted for age (*P* for interaction < 0.001). Subgroup analyses by age revealed that overweight and obese individuals with T2DM had a progressive increase in AIP, but the trend of increasing risk of AIP-associated overweight and obese T2DM slowed as age increased (Fig. 5). Specifically, the risk of T2DM increased by a factor of 9.10 for each unit increase in AIP in overweight and obese participants younger than 40 years, which was 5.46-fold greater than the risk of T2DM for individuals aged 60 years and older (Fig. 4).

### Inflammatory cells involved in the effects of AIP on overweight and obese individuals with T2DM

The present study employed mediation analysis to investigate the role of inflammatory cells in mediating the association between the AIP and overweight/obesity-related T2DM. Table 5 demonstrates that lymphocytes, monocytes, eosinophils, neutrophils, and WBC significantly mediated the association between AIP and T2DM in overweight and obese individuals, with the exception of basophils (*P* = 0.334). Notably, neutrophils, WBC, and monocytes had the most substantial mediating effects, with mediating ratios of 4.66%, 4.16%, and 1.93%, respectively.

**Table 5:** The mediating effects of inflammatory factors on the association between AIP and risk of T2DM

Inflammatory factors	Indirect effects	Direct effects	Total effects		Mediated proportion (%)	*P* value
	OR (95% CI)	OR (95% CI)	OR (95% CI)			
Lymphocytes	0.09e-02 (0.05e-02, 0.15e-02)^*^	9.00e-02 (8.43e-02, 9.53e-02)^*^	9.09e-02 (8.54e-02, 9.63e-02)^*^		1.03	< 0.001^*^
Monocytes	0.18e-02 (0.13e-02, 0.23e-02)^*^	8.90e-02 (8.35e-02, 9.46e-02)^*^	9.10e-02 (8.54e-02, 9.63e-02)^*^		1.93	< 0.001^*^
Eosinophils	0.02e-02 (0.01e-02, 0.03e-02)^*^	9.08e-02 (8.52e-02, 9.42e-02)^*^	9.09e-02 (8.53e-02, 9.63e-02)^*^		0.19	0.002^*^
Basophils	0.01e-02 (-0.01e-02, 0.03e-02)^*^	9.07e-02 (8.52e-02, 9.62e-02)^*^	9.07e-02 (8.52e-02, 9.62e-02)^*^		0.10	0.334
Neutrophils	0.38e-02 (0.32e-02, 0.44e-02)^*^	8.73e-02 (8.12e-02, 9.26e-02)^*^	9.11e-02 (8.55e-02, 9.64e-02)^*^		4.16	< 0.001^*^
WBC	0.42e-02 (0.36e-02, 0.50e-02)^*^	8.69e-02 (8.13e-02, 9.22e-02)^*^	9.11e-02 (8.55e-02, 9.64e-02)^*^		4.66	< 0.001^*^

*P < 0.05

All covariates including age, sex, occupation, BMI, hypertension, total protein, total bilirubin, ALT, AST, GGT, ALP, Cre, and UA were adjusted in this model

*CI* confidence interval, *WBC* white blood cell

## Discussion

In this investigation involving participants undergoing health screenings, we detected a significant correlation between elevated AIP levels and an increased risk of T2DM in overweight and obese individuals, even after accounting for confounding factors. Furthermore, we observed a declining association between AIP and overweight/obese-related T2DM as age increased, with the strongest association identified among adults under 40 years old. Notably, we discovered a J-shaped curve in the association between AIP and overweight/obese-related T2DM, with a turning point at − 0.07. The mediation analysis revealed that inflammatory cells played a mediating role in this association. These findings carry substantial implications for the prevention and management of T2DM in overweight and obese individuals.

Previous studies have consistently demonstrated that atherogenic dyslipidemia, characterized by elevated TG levels and reduced HDL-C levels, is a prominent feature in diabetes-prone environments or individuals with diabetes [25]. A prospective study conducted on the Kailuan cohort, comprising 52,225 Chinese adult participants, elucidated a significant, independent association between cumulative AIP and the risk of T2DM after adjusting for confounding factors. This association remained consistent across various subgroups based on age, sex, medication use, and blood pressure, highlighting the potential of AIP as a tool for identifying individuals at risk for T2DM [26]. Similarly, a secondary analysis of the China Health and Retirement Longitudinal Study evaluated the predictive value of changes in AIP between baseline and follow-up assessments for the development of T2DM. The results indicated that sustained high AIP levels, transitions from high to low AIP levels, and transitions from low to high AIP levels were all associated with the occurrence of T2DM in middle-aged and elderly Chinese individuals [27]. Collectively, these comprehensive findings suggest that AIP holds promise as a useful indicator for identifying individuals with T2DM.

This study specifically focused on overweight and obese individuals with the aim of investigating the association between the AIP and T2DM. The study also took into consideration the impact of obesity on the incidence of diabetes. The results indicate that overweight and obesity in individuals with T2DM are independently associated with the AIP and demonstrate a non-linear correlation. Notably, a significant increase in the association between overweight, obesity, and diabetes was observed when the AIP > -0.07, with an OR of 5.71. Previous research has shown that mean AIP values in the general population range from − 0.24 to 0.55 [7], with AIP values below 0.11 considered low risk [28].

Furthermore, a recent cross-sectional study based on the NHANES database, comprising 9245 American adults, revealed a J-shaped association between AIP and T2DM after adjusting for confounding factors. Specifically, an increase in the AIP value beyond − 0.47 was significantly correlated with an increased risk of T2DM, with an OR of 5.39 [11]. Another analysis, using data from the Action to Control Cardiovascular Risk in Diabetes study that included 10,251 participants, demonstrated a cutoff value of 0.34 for the AIP associated with T2DM, which aligns with the findings of the present study [29]. Consistent with previous research, individuals with dyslipidemia have consistently shown a higher risk of developing T2DM [30]. TGs are the most abundant lipids in human adipose tissue, and elevated TG levels can lead to biotoxicity, further contributing to the development and progression of insulin resistance [31]. On the other hand, HDL-C, which contains various lipids and proteins, plays a crucial role in regulating metabolic diseases through its antioxidant and anti-inflammatory functions [32]. The AIP, which combines TG and HDL-C levels, not only reflects the ratio of TG to HDL-C but also the size of lipoprotein particles. Consequently, the AIP provides a more comprehensive indication of the pathogenicity and specificity of lipid abnormalities when compared to high TG or low HDL-C levels alone [33]. Additionally, it is widely recognized that weight gain plays a pivotal role in the incidence of diabetes. A prospective study involving a Chinese multicenter population conducted by Zhang et al. discovered that overweight and obesity are significant risk factors for impaired glucose tolerance [34]. In the onset of T2DM, pancreatic β-cells struggle to cope with the increased insulin demand caused by insulin resistance. Overweight and obesity often lead to nutrient overload, resulting in the release of reactive oxygen species and subsequent oxidative stress [35]. This, in turn, further contributes to an increased influx of endoplasmic reticulum stress, exacerbating the toxic effects of lipids on β-cells [36]. Therefore, the AIP may serve as a more predictive indicator for the risk of developing T2DM in overweight and obese individuals.

Furthermore, the findings of this study revealed that the association between the AIP and T2DM remains consistent across various subgroup analyses, independent of age. However, when the subgroup analyses were categorized by age, it was observed that the association between overweight and obese individuals with T2DM and the AIP increased progressively with age, whereas the association between the AIP and overweight and obese individuals with T2DM decreased with age. Additionally, the smooth curve fitting analysis demonstrated different curve shapes for the AIP and T2DM risk at different age groups.

Age is widely recognized as a significant risk factor for diabetes, with previous research indicating that the prevalence of diabetes in China rises with increasing age [37]. Older individuals and those who are obese are at a higher risk of developing T2DM [38]. In comparison to older participants, younger individuals may have dietary preferences that promote inflammation [39], experience higher stress levels, and exhibit depressive symptoms [40], all of which can heighten the risk of T2DM. Existing evidence suggests that impaired glycemic control in young individuals is associated with both biological and psychological factors, including poor dietary choices, sedentary behavior, adverse life events, chronic stress, and increased levels of depression [41]. Furthermore, younger participants may demonstrate a lesser level of concern regarding lipid abnormalities. It is worth noting that lipid-lowering therapy decreases the AIP [42], and younger individuals with lipid abnormalities are less likely to receive lipid-lowering therapy [43]. This discrepancy may result in lipid imbalance and potentially amplify the association between the AIP and T2DM. On the other hand, older individuals have a higher probability of receiving lipid-lowering therapy, which could subsequently weaken the potential association between the AIP and T2DM risk [44].

In line with our findings, a cohort study conducted by Li among 7670 individuals from the general population in Taiwan Province demonstrated that the risk associated with the AIP for T2DM was lower in participants aged 40–64 years compared to those younger than 20 years [10]. Moreover, a recent real-world prospective cohort study involving 42,925 general participants indicated that the risk of T2DM associated with the AIP and cumulative inflammation is highly dependent on age, with the highest risk observed in adults younger than 40 years and a significant decrease in risk as age advances [45]. In contrast, another study involving adult participants from the NHANES suggested that the association between the AIP and T2DM does not vary by age [46]. This discrepancy could potentially be attributed to differences in the population samples selected for the respective studies.

Several cellular processes have been identified in the pathogenesis of T2DM. Research has demonstrated that inflammation plays a crucial role in the development of T2DM [47, 48]. Individuals with obesity-related diabetes are known to exhibit a state of inflammation [49]. Our research has revealed an increase in various inflammatory cells in T2DM patients, with neutrophils, leukocytes, and monocytes exhibiting the most significant mediating effects on inflammation associated with T2DM. Studies have highlighted the importance of neutrophils as one of the key peripheral cells in diabetes [50]. These cells primarily produce an excess of superoxide, cytokines, and tumor necrosis factors that promote the inflammatory state in diabetes [51]. Animal studies have demonstrated that blocking neutrophil activity in the early stages can limit the progression of diabetes in mice [52]. Additionally, Chatterjee and colleagues reported that a high leukocyte count leads to dysregulated blood glucose levels, reducing the likelihood of restoring normal glucose levels [53]. Recent studies have indicated that an increased leukocyte count is associated with the onset of T2DM, and a BMI greater than 25 kg/m^2^ may mediate this association [54]. Gu et al. reported that the leukocyte count could serve as an indicator of whether obesity increases the risk of developing diabetes [55]. Importantly, the role of monocytes is considered a significant characteristic of diabetes [56]. Monocytes contribute to the development of T2DM and cardiovascular diseases by mediating the effects of resistin [57]. These findings support the conclusion of this study, which demonstrates that inflammatory cells participate in the development of diabetes induced by lipid abnormalities through multiple mechanisms.

### Limitations and strengths

The strengths of this study include the utilization of Chinese national standards for classifying obesity and the incorporation of a large sample size, which provided robust statistical support for the adjusted logistic regression analysis. Furthermore, this study addresses a research gap by examining the correlation between the AIP and T2DM risk in individuals with a BMI ≥ 24 kg/m^2^. Subgroup analyses and interaction tests were conducted to explore the associations between AIP and T2DM in overweight and obese patients, yielding valuable insights for diabetes prevention and screening in different population subgroups. However, it is important to acknowledge the limitations of this study. First, due to its cross-sectional design, establishing a causal association between AIP and T2DM in overweight and obese patients was not feasible. Second, even though efforts were made to collect comprehensive data on confounding variables, there may still be unadjusted variables that could introduce residual confounders, such as smoking, alcohol consumption, and lipid-lowering therapy program. Additionally, due to limitations of the health screening program, additional inflammatory factors (e.g., (hs)CRP) were not collected in this study for mediated effect assessment. Finally, this single-center study focused on a specific health screening population, limiting the generalizability of the findings to other groups. These limitations emphasize the necessity for further research to comprehend the factors influencing diabetes.

## Conclusion

Current research suggests that there is an independent association between AIP and an increased risk of T2DM. The risk of developing T2DM significantly rises when the AIP is greater than − 0.07 and this association weakens progressively with age. The AIP has been validated as a reliable marker for identifying individuals at risk for T2DM, especially those under the age of 40. Furthermore, inflammatory cells have been identified as potential mediators of the association between AIP and T2DM among overweight and obese individuals. Additional prospective studies are needed to confirm our findings beyond the population undergoing health screenings.

### Electronic supplementary material

Below is the link to the electronic supplementary material.

Supplementary Material 1

## Data Availability

Contact the first author for all data relating to this study on reasonable request.

## References

[1] Ahmad E, Lim S, Lamptey R, Webb DR, Davies MJ (2022). Type 2 diabetes. Lancet.

[2] Ong KL, Stafford LK, McLaughlin SA, Boyko EJ, Vollset SE, Smith AE, Dalton BE, Duprey J, Cruz JA, Hagins H, Lindstedt PA (2023). Global regional, national burden of diabetes: from 1990 to 2021, with projections of prevalence to 2050: a systematic analysis for the global burden of disease study 2021. Lancet..

[3] Sun H, Saeedi P, Karuranga S, Pinkepank M, Ogurtsova K, Duncan BB, Stein C, Basit A, Chan JCN, Mbanya JC (2022). IDF diabetes atlas: global, regional and country-level diabetes prevalence estimates for 2021 and projections for 2045. Diabetes Res Clin Pract.

[4] Magliano DJ, Boyko EJ. Committee IDFDAtes: IDF Diabetes Atlas. Idf Diabetes Atlas. Brussels: International Diabetes Federation © International Diabetes Federation; 2021.

[5] Zheng X, Zhang X, Han Y, Hu H, Cao C (2023). Nonlinear relationship between atherogenic index of plasma and the risk of prediabetes: a retrospective study based on Chinese adults. Cardiovasc Diabetol.

[6] Won KB, Heo R, Park HB, Lee BK, Lin FY, Hadamitzky M, Kim YJ, Sung JM, Conte E, Andreini D (2021). Atherogenic index of plasma and the risk of rapid progression of coronary atherosclerosis beyond traditional risk factors. Atherosclerosis.

[7] Dobiásová M, Frohlich J (2001). The plasma parameter log (TG/HDL-C) as an atherogenic index: correlation with lipoprotein particle size and esterification rate in apob-lipoprotein-depleted plasma (FER(HDL)). Clin Biochem.

[8] Tan M, Zhang Y, Jin L, Wang Y, Cui W, Nasifu L, He B (2023). Association between atherogenic index of plasma and prehypertension or hypertension among normoglycemia subjects in a Japan population: a cross-sectional study. Lipids Health Dis.

[9] Zhu X, Yu L, Zhou H, Ma Q, Zhou X, Lei T, Hu J, Xu W, Yi N, Lei S (2018). Atherogenic index of plasma is a novel and better biomarker associated with obesity: a population-based cross-sectional study in China. Lipids Health Dis.

[10] Li YW, Kao TW, Chang PK, Chen WL, Wu LW (2021). Atherogenic index of plasma as predictors for metabolic syndrome, hypertension and diabetes mellitus in Taiwan citizens: a 9-year longitudinal study. Sci Rep.

[11] Yin B, Wu Z, Xia Y, Xiao S, Chen L, Li Y (2023). Non-linear association of atherogenic index of plasma with insulin resistance and type 2 diabetes: a cross-sectional study. Cardiovasc Diabetol.

[12] Qin Z, Zhou K, Li Y, Cheng W, Wang Z, Wang J, Gao F, Yang L, Xu Y, Wu Y (2020). The atherogenic index of plasma plays an important role in predicting the prognosis of type 2 diabetic subjects undergoing percutaneous coronary intervention: results from an observational cohort study in China. Cardiovasc Diabetol.

[13] Wu X, Qiu W, Yang H, Chen YJ, Liu J, Zhao G (2024). Associations of the triglyceride-glucose index and atherogenic index of plasma with the severity of new-onset coronary artery disease in different glucose metabolic states. Cardiovasc Diabetol.

[14] Lin J, Li H, Wan Q (2022). A cross-sectional study of the correlation between the Atherogenic Index of plasma and nonalcoholic fatty liver disease in patients with type 2 diabetes. Diabetes Metab Syndr Obes: Targets Ther.

[15] Neeland IJ, Ross R, Després JP, Matsuzawa Y, Yamashita S, Shai I, Seidell J, Magni P, Santos RD, Arsenault B (2019). Visceral and ectopic fat, atherosclerosis, and cardiometabolic disease: a position statement. Lancet Diabetes Endocrinol.

[16] Lee SW, Son JY, Kim JM, Hwang SS, Han JS, Heo NJ (2018). Body fat distribution is more predictive of all-cause mortality than overall adiposity. Diabetes Obes Metab.

[17] Hansen SEJ, Madsen CM, Varbo A, Nordestgaard BG (2019). Low-Grade inflammation in the association between mild-to-moderate hypertriglyceridemia and risk of Acute Pancreatitis: a study of more than 115000 individuals from the General Population. Clin Chem.

[18] Eguchi K, Nagai R (2017). Islet inflammation in type 2 diabetes and physiology. J Clin Investig.

[19] Bertoni AG, Burke GL, Owusu JA, Carnethon MR, Vaidya D, Barr RG, Jenny NS, Ouyang P, Rotter JI (2010). Inflammation and the incidence of type 2 diabetes: the multi-ethnic study of atherosclerosis (MESA). Diabetes Care.

[20] Saltiel AR, Olefsky JM (2017). Inflammatory mechanisms linking obesity and metabolic disease. J Clin Investig.

[21] American Diabetes Association (2013). Standards of medical care in diabetes-2013. Diabetes Care.

[22] Zhou BF (2002). Predictive values of body mass index and waist circumference for risk factors of certain related diseases in Chinese adults–study on optimal cut-off points of body mass index and waist circumference in Chinese adults. Biomed Environ Sci: BES.

[23] Zhang M, Shi Y, Zhou B, Huang Z, Zhao Z, Li C, Zhang X, Han G, Peng K, Li X (2023). Prevalence, awareness, treatment, and control of hypertension in China, 2004-18: findings from six rounds of a national survey. BMJ (Clinical Res ed).

[24] Sun C, Xiao X, Yan L, Sheng L, Wang Q, Jiang P, Lian M, Li Y, Wei Y, Zhang J (2019). Histologically proven AMA positive primary biliary cholangitis but normal serum alkaline phosphatase: is alkaline phosphatase truly a surrogate marker?. J Autoimmun.

[25] Jakubiak GK, Cieślar G, Stanek A, Nitrotyrosine (2022). Nitrated lipoproteins, and cardiovascular dysfunction in patients with type 2 diabetes: what do we know and what remains to be explained?. Antioxidants (Basel, Switzerland).

[26] Lan Y, Chen G, Wu D, Ding X, Huang Z, Wang X, Balmer L, Li X, Song M, Wang W (2023). Temporal relationship between atherogenic dyslipidemia and inflammation and their joint cumulative effect on type 2 diabetes onset: a longitudinal cohort study. BMC Med.

[27] Yi Q, Ren Z, Bai G, Zhu S, Li S, Li C, Wu H, Zhu Y, Song P (2022). The longitudinal effect of the atherogenic index of plasma on type 2 diabetes in middle-aged and older Chinese. Acta Diabetol.

[28] Salazar MR, Carbajal HA, Espeche WG, Aizpurúa M, Leiva Sisnieguez CE, March CE, Balbín E, Stavile RN, Reaven GM (2013). Identifying cardiovascular disease risk and outcome: use of the plasma triglyceride/high-density lipoprotein cholesterol concentration ratio versus metabolic syndrome criteria. J Intern Med.

[29] Fu L, Zhou Y, Sun J, Zhu Z, Xing Z, Zhou S, Wang Y, Tai S (2021). Atherogenic index of plasma is associated with major adverse cardiovascular events in patients with type 2 diabetes mellitus. Cardiovasc Diabetol.

[30] Peng J, Zhao F, Yang X, Pan X, Xin J, Wu M, Peng YG (2021). Association between dyslipidemia and risk of type 2 diabetes mellitus in middle-aged and older Chinese adults: a secondary analysis of a nationwide cohort. BMJ open.

[31] Manell H, Kristinsson H, Kullberg J, Ubhayasekera SJK, Mörwald K, Staaf J, Cadamuro J, Zsoldos F, Göpel S, Sargsyan E (2019). Hyperglucagonemia in youth is associated with high plasma free fatty acids, visceral adiposity, and impaired glucose tolerance. Pediatr Diabetes.

[32] Di Bartolo BA, Cartland SP, Genner S, Manuneedhi Cholan P, Vellozzi M, Rye KA, Kavurma MM (2021). HDL improves cholesterol and glucose homeostasis and reduces atherosclerosis in diabetes-associated atherosclerosis. J Diabetes Res.

[33] Fernández-Macías JC, Ochoa-Martínez AC, Varela-Silva JA, Pérez-Maldonado IN (2019). Atherogenic index of plasma: novel predictive biomarker for cardiovascular illnesses. Arch Med Res.

[34] Zhang X, Yue Y, Liu S, Cong X, Wang W, Li J (2023). Relationship between BMI and risk of impaired glucose tolerance and impaired fasting glucose in Chinese adults: a prospective study. BMC Public Health.

[35] Houstis N, Rosen ED, Lander ES (2006). Reactive oxygen species have a causal role in multiple forms of insulin resistance. Nature.

[36] Unger RH (1995). Lipotoxicity in the pathogenesis of obesity-dependent NIDDM: genetic and clinical implications. Diabetes.

[37] Liang D, Fan G (2020). Social support and user characteristics in online diabetes communities: an in-depth survey of a large-scale Chinese population. Int J Environ Res Public Health.

[38] Kirkman MS, Briscoe VJ, Clark N, Florez H, Haas LB, Halter JB, Huang ES, Korytkowski MT, Munshi MN, Odegard PS (2012). Diabetes in older adults. Diabetes Care.

[39] Shivakoti R, Biggs ML, Djoussé L, Durda PJ, Kizer JR, Psaty B, Reiner AP, Tracy RP, Siscovick D, Mukamal KJ (2022). Intake and sources of dietary fiber, inflammation, and cardiovascular disease in older US adults. JAMA Netw Open.

[40] Latham RM, Kieling C, Arseneault L, Kohrt BA, Moffitt TE, Rasmussen LJH, Rocha TB, Mondelli V, Fisher HL (2022). Longitudinal associations between adolescents' individualised risk for depression and inflammation in a UK cohort study.. Brain, Behavior, Immun.

[41] Hessler DM, Fisher L, Mullan JT, Glasgow RE, Masharani U (2011). Patient age: a neglected factor when considering disease management in adults with type 2 diabetes. Patient Educ Couns.

[42] Safari S, Amini M, Aminorroaya A, Feizi A (2020). Patterns of changes in serum lipid profiles in prediabetic subjects: results from a 16-year prospective cohort study among first-degree relatives of type 2 diabetic patients. Lipids Health Dis.

[43] Na E, Cho S, Kim DJ, Choi J, Han E (2020). Time-varying and dose-dependent effect of long-term statin use on risk of type 2 diabetes: a retrospective cohort study. Cardiovasc Diabetol.

[44] Kleipool EE, Dorresteijn JA, Smulders YM, Visseren FL, Peters MJ, Muller M (2020). Treatment of hypercholesterolaemia in older adults calls for a patient-centred approach. Heart.

[45] Lan Y, Wu D, Cai Z, Xu Y, Ding X, Wu W, Lan S, Chen L, Guo Z, Balmer L (2023). Supra-additive effect of chronic inflammation and atherogenic dyslipidemia on developing type 2 diabetes among young adults: a prospective cohort study. Cardiovasc Diabetol.

[46] Shi Y, Wen M (2023). Sex-specific differences in the effect of the atherogenic index of plasma on prediabetes and diabetes in the NHANES 2011–2018 population. Cardiovasc Diabetol.

[47] Dubreuil M, Rho YH, Man A, Zhu Y, Zhang Y, Love TJ, Ogdie A, Gelfand JM, Choi HK (2014). Diabetes incidence in psoriatic arthritis, psoriasis and rheumatoid arthritis: a UK population-based cohort study. Rheumatology (Oxford).

[48] Huang M, Liu F, Li Z, Liu Y, Su J, Ma M, He Y, Bu H, Gao S, Wang H (2023). Relationship between red cell distribution width/albumin ratio and carotid plaque in different glucose metabolic states in patients with coronary heart disease: a RCSCD-TCM study in China. Cardiovasc Diabetol.

[49] Wu D, Lan Y, Chen S, Ding X, Chen G, Wu C, Balmer L, Xu W, Wu S, Wang W (2023). Combined effect of adiposity and elevated inflammation on incident type 2 diabetes: a prospective cohort study. Cardiovasc Diabetol.

[50] Xiao Z, Shen D, Lan T, Wei C, Wu W, Sun Q, Luo Z, Chen W, Zhang Y, Hu L (2022). Reduction of lactoferrin aggravates neuronal ferroptosis after intracerebral hemorrhagic stroke in hyperglycemic mice. Redox Biol.

[51] Das SK, Yuan YF, Li MQ (2018). Specific PKC βII inhibitor: one stone two birds in the treatment of diabetic foot ulcers. Biosci Rep.

[52] Diana J, Simoni Y, Furio L, Beaudoin L, Agerberth B, Barrat F, Lehuen A (2013). Crosstalk between neutrophils, B-1a cells and plasmacytoid dendritic cells initiates autoimmune diabetes. Nat Med.

[53] Chatterjee R, Kwee LC, Pagidipati N, Koweek LH, Mettu PS, Haddad F, Maron DJ, Rodriguez F, Mega JL, Hernandez A (2022). Multi-dimensional characterization of prediabetes in the Project Baseline Health Study. Cardiovasc Diabetol.

[54] Hsieh CY, Lee WH, Liu YH, Lu CC, Chen SC, Su HM (2023). Significant impact of body mass index on the relationship between increased white blood cell count and new-onset diabetes. Int J Med Sci.

[55] Gu Y, Hu K, Huang Y, Zhang Q, Liu L, Meng G, Wu H, Xia Y, Bao X, Shi H (2018). White blood cells count as an indicator to identify whether obesity leads to increased risk of type 2 diabetes. Diabetes Res Clin Pract.

[56] Clarke MC, Figg N, Maguire JJ, Davenport AP, Goddard M, Littlewood TD, Bennett MR (2006). Apoptosis of vascular smooth muscle cells induces features of plaque vulnerability in atherosclerosis. Nat Med.

[57] Alfhili MA, Alsughayyir J, Basudan A, Alfaifi M, Awan ZA, Algethami MR, Al-Sheikh YA (2023). Blood indices of omega-3 and omega-6 polyunsaturated fatty acids are altered in hyperglycemia. Saudi J Biol Sci.

