# Supplementary material for: Nonlinear association between atherogenic index of plasma and type 2 diabetes mellitus in overweight and obesity patients: evidence from Chinese medical examination data
Source: Cardiovasc Diabetol. 2024 Jun 29;23:226. doi: 10.1186/s12933-024-02330-y (PMC11218131; doi:10.1186/s12933-024-02330-y)
Supplement: Supplementary file 1 — Supplementary Material 1 [file 12933_2024_2330_MOESM1_ESM.docx]

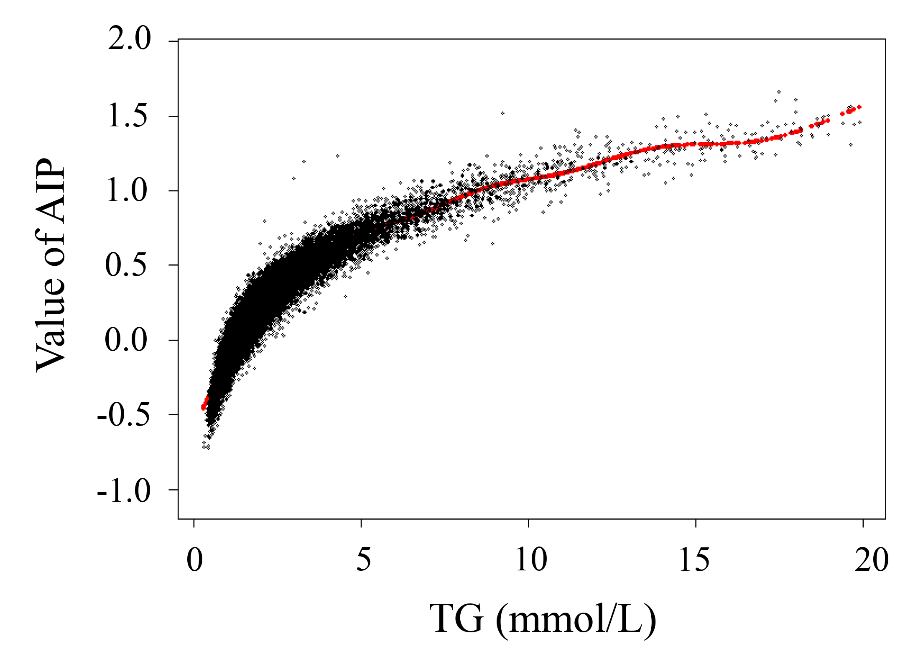


SFig.1 Generalized additive model with fitting smoothness for the dose–response relationship between TG and AIP. The black dots in the figure indicate each specific data sample point, showing the AIP values for different TG values, and the density of the data points shows the distribution of the samples. The red smoothed curves indicate the results of smoothing and fitting the data through the generalized additive model (GAM), and the curves show the overall trend between TG and AIP. AIP, atherogenic index of plasma.


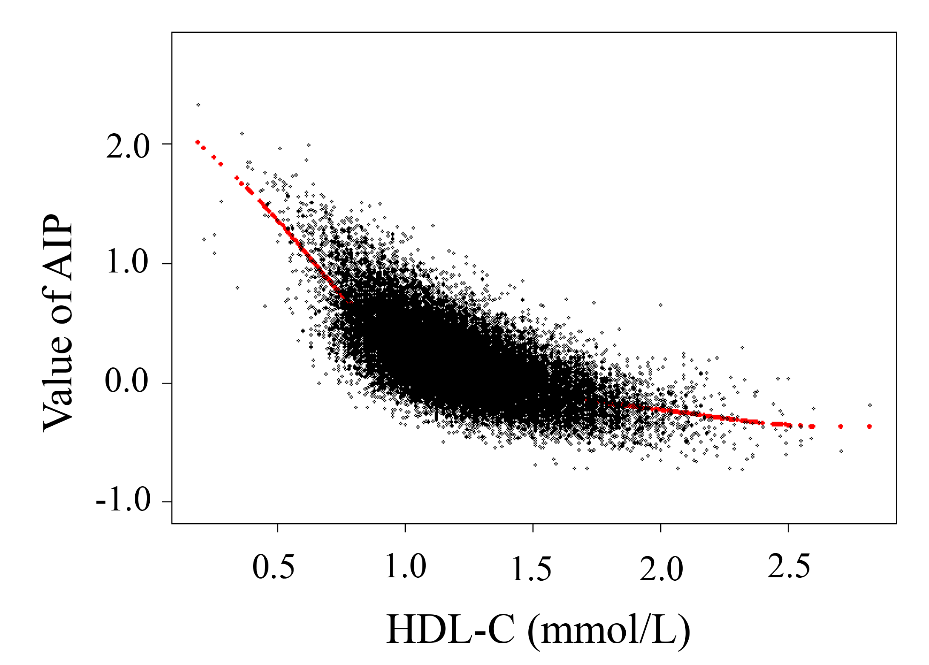


SFig.2 Generalized additive model with fitting smoothness for the dose–response relationship between HDL-C and AIP. The black dots in the figure indicate each specific data sample point, showing the AIP values for different HDL-C values, and the density of the data points shows the distribution of the samples. The red smoothed curves indicate the results of smoothing and fitting the data through the generalized additive model (GAM), and the curves show the overall trend between HDL-C and AIP. AIP, atherogenic index of plasma.
